# Computational fluid dynamics analysis for enlargement of the descending aorta after frozen elephant trunk implantation

**DOI:** 10.1186/s13019-026-04095-9

**Published:** 2026-04-18

**Authors:** Hiroki Nakabori, Kenji Iino, Ai Sakai, Masaki Kitazawa, Hideyasu Ueda, Yoshitaka Yamamoto, Yukiko Yamada, Akira Murata, Hirofumi Takemura, Takahiro Kiwata

**Affiliations:** 1 Department of Cardiovascular Surgery, Kanazawa University, 13-1 Takaramachi, Kanazawa, 920-8641 Japan; 2 School of Mechanical Engineering, Kanazawa University, Kanazawa, Japan

**Keywords:** Computational fluid dynamics, Frozen elephant trunk, Aortic dissection, Wall shear stress, Oscillatory shear index

## Abstract

**Background:**

Acute type A aortic dissection (ATAAD) treated with total arch replacement (TAR) plus frozen elephant trunk (FET) can achieve favorable early remodeling; however, late enlargement of the distal descending aorta still occurs in some patients.

**Methods:**

Study 1 included ATAAD patients who underwent TAR with FET between January 2015 and December 2023 and achieved complete false-lumen obliteration at the distal FET level. We identified cases with more than 5 mm enlargement at the distal FET level during follow-up and performed statistical analyses. Among these, patients with contrast-enhanced CT were analyzed using CFD. Study 2 used idealized aorta–stent graft models with different aorto-stent graft angles (AS angle) to isolate the hemodynamic effect of angulation.

**Results:**

In Study 1, larger AS angle was associated with subsequent aortic enlargement, and a cutoff around 38.3° suggested higher risk. CFD tended to show disturbed/recirculating flow along the lesser curvature in high-angle cases, with lower WSS and higher OSI at the distal FET level. In Study 2, increasing AS angle accentuated lesser-curvature flow disturbance and increased greater-curvature WSS; Pearson correlations suggested a positive association between AS angle and greater-curvature WSS/TAWSS.

**Conclusions:**

A larger AS angle may be associated with enlargement of the distal aorta after FET. Larger and more precise studies are warranted to validate these findings.

**Supplementary Information:**

The online version contains supplementary material available at 10.1186/s13019-026-04095-9.

## Introduction

Acute Stanford type A aortic dissection (ATAAD) is a lethal acute aortic syndrome involving the ascending aorta, with untreated mortality increasing hour by hour [1]. Therefore, current guidelines recommend emergent surgery and, where feasible, referral to high-volume aortic centers [2, 3]. The main goals of surgery are closure of the proximal entry tear, control of proximal aortic rupture, and restoration of true-lumen flow to improve end-organ perfusion. Surgical strategies range from ascending/hemiarch replacement to total arch replacement (TAR). In cases with extensive arch–proximal descending dissection or when distal disease control is required, TAR combined with a frozen elephant trunk (FET) is often used. FET can proximalize the distal anastomosis while reducing false-lumen pressurization in the proximal descending aorta, promoting false-lumen thrombosis and distal remodeling [4, 5]. Several FET devices are available, including the E-vita Open/Open Plus (Jotec/CryoLife, Kennesaw, GA, USA) and Thoraflex Hybrid (Terumo Aortic, FL, USA) [6]. In Japan, the FROZENIX^®^ J Graft Open Stent Graft (Japan Lifeline, Tokyo, Japan) has been available since 2014, and favorable outcomes have been reported [7–9]. However, extended arch surgery with FET can be associated with severe complications such as stroke and spinal cord ischemia [10]. FET-specific late issues include distal stent graft–induced new entry (dSINE) [11] and late aortic enlargement (negative remodeling) [12]. Hemodynamic mechanisms of dSINE have been investigated using CFD [13–15] and discussed in conjunction with mechanical factors at the stent-graft edge [16]. For negative remodeling, associations have been suggested with false-lumen thrombosis and the number of residual entries [17], as well as with patient factors such as race [18] and ulcer-like projection (ULP) [19]. Because multiple factors contribute, the detailed mechanism of late enlargement remains incompletely understood [12]. Zhu et al. highlighted true–false lumen pressure differences and reported CFD-based prediction of late enlargement [20].

Anatomical factors such as aortic size and lumen configuration are important predictors of disease progression, but recent studies suggest that aortic hemodynamic parameters may also be independent prognostic markers [21]. In routine postoperative follow-up, contrast-enhanced CT primarily evaluates anatomy (true/false lumen configuration, flap morphology, etc.) and does not directly measure time-resolved hemodynamics. Accordingly, 4D-flow MRI and computational fluid dynamics (CFD) have been used to quantify hemodynamic indices [22]. CFD enables blood-flow simulation in patient-specific 3D models and allows assessment of streamlines, wall shear stress (WSS), time-averaged wall shear stress (TAWSS), and oscillatory shear index (OSI) [23].

In aortic aneurysm and dissection, abnormal flow patterns such as vortical and helical flow are frequently observed on streamline visualization and are linked to disturbed hemodynamics [24]. Takehara et al. concluded that disturbed vortex/helical flow is reflected by low WSS and high OSI [25]. The combination of low WSS and high OSI has been implicated in aneurysm growth and rupture [26]. In addition, the physiological range of WSS on normal arterial walls has been reported to be approximately 1–7 Pa, and deviations from this range may trigger pathological processes [27]; excessive shear stress may also injure the endothelium [28].

Based on these studies, we hypothesized that post-FET hemodynamic changes contribute to negative remodeling. Specifically, an excessively acute stent-graft angulation may direct the distal graft outflow toward the aortic wall rather than downstream, potentially producing high WSS along the greater curvature, while relative narrowing distal to the graft may promote flow disturbance along the lesser curvature, creating regions of low WSS and high OSI. We therefore investigated whether a steep aorto–stent graft angle is associated with negative remodeling. Among 117 ATAAD patients treated with TAR plus FET, we selected 47 patients with favorable early remodeling and evaluated the association between stent-graft angulation and subsequent negative remodeling using statistical analyses. Among these, 24 patients with contrast-enhanced CT underwent CFD to explore hemodynamic mechanisms. We also created idealized models focused on the stent-graft angle to isolate the hemodynamic effects of angulation from patient-related variables.

## Methods

### Study design

#### Study 1. Patient-specific statistical analysis and CFD (patient-specific models)

**Fig. 1:**
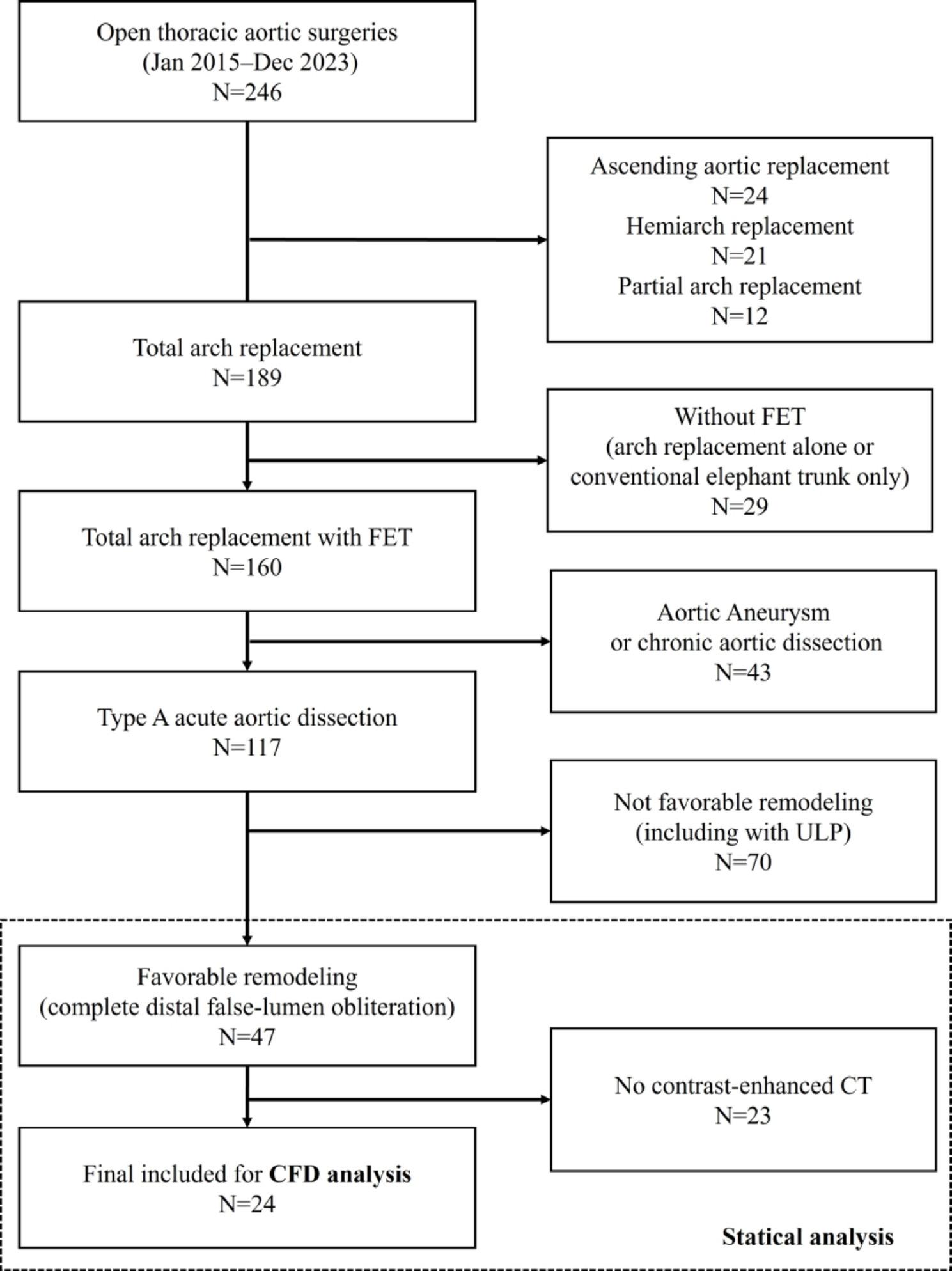
Study 1 flowchart. Among 246 open aortic surgeries, 117 ATAAD patients underwent TAR with FET. Successful remodeling (complete false-lumen obliteration at the distal FET level) was identified in 47 patients for statistical analysis; 24 with contrast-enhanced CT were included for CFD

**Fig. 2:**
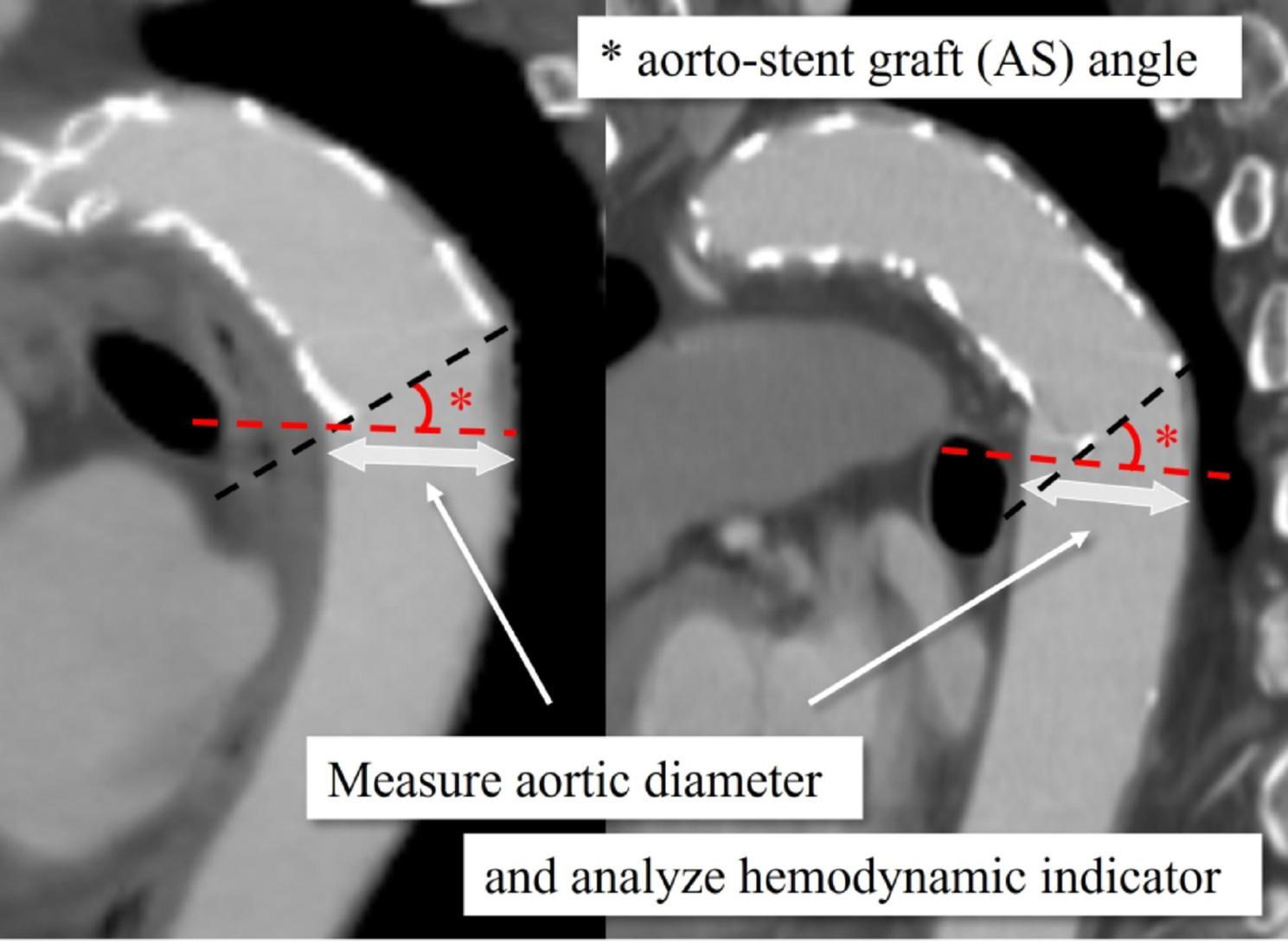
Definition of AS angle and measurement location. AS angle was defined as the angle between the aortic plane through the distal stent-graft end (red dashed line) and the distal stent-graft axis (black dashed line), shown by the asterisk. Aortic diameter change was measured as the difference in minor-axis diameter on the distal FET plane between the first follow-up confirming remodeling and the latest follow-up CT (white double arrow)

The Medical Ethics Committee of Kanazawa University (Approval No. 2022 − 105 [114070]) approved this study. Among 246 open aortic procedures performed at our institution between January 2015 and December 2023, 117 patients underwent TAR with FET for ATAAD. We defined “successful remodeling” as complete obliteration of the false lumen at the distal FET level on follow-up CT (qualitative imaging assessment) and identified 47 patients for statistical analysis (Fig. 1). During the early phase of FET adoption (around 2015), some TAR cases were completed with arch replacement alone or a conventional elephant trunk; however, in our institution, FET has been routinely used for cases requiring TAR. Operative details have been reported previously [29]. To eliminate the influence of distal re-entry, we included only cases in which the false lumen was completely obliterated; patients in whom the distal FET landed within a pre-existing aneurysmal sac were excluded. We defined the aorto–stent graft angle (AS angle) as the angle between the aortic plane passing through the distal end of the stent graft (red dashed line in Fig. 2) and the distal end of the stent graft (black dashed line in Fig. 2). Aortic diameter change was assessed using the minor-axis diameter on the cross-sectional plane passing through the distal stent-graft end (white double arrow in Fig. 2), comparing the first follow-up CT confirming successful remodeling with the most recent follow-up CT. Following prior studies, we defined “enlargement” as an increase in minor-axis diameter more than 5 mm [30]. We compared enlargement and non-enlargement groups and generated a receiver operating characteristic (ROC) curve to evaluate the association between AS angle (measured after remodeling) and subsequent enlargement.

Finally, from these 47 successfully remodeled patients, those without contrast-enhanced CT data were excluded, leaving 24 patients for CFD analysis. In our routine follow-up protocol (non–ECG-gated CT), CT is typically performed within 1 week to 1 month after surgery, then at 3 months and 6 months during the first postoperative year, and every 6–12 months thereafter; these CT datasets were used for the present analyses.

#### Study 2: CFD analysis(Idealized models)

To simulate a stent graft within the aorta, a 3D model was created. The AS angles included 0°, 20°, 40°, and 60°, and CFD analyses were performed for each configuration. To ensure that AS angle remained the sole variable, the distal cross-sectional area of the stent graft was kept constant across the models.

### Geometry and meshing

Study 1. CT scans were obtained with a 256-slice scanner (Revolution CT, GE Healthcare, Waukesha, WI, USA) and saved in Digital Imaging and Communications in Medicine (DICOM) format. DICOM data were imported into RadiAnt DICOM Viewer (v2023.1; Medixant, Poznań, Poland). The aortic lumen was manually segmented on the reconstructed 3D dataset after removing surrounding structures. The extraction region was selected from the proximal end of the FET to the diaphragmatic level to sufficiently include the thoracic descending aorta distal to the stent graft; arch branch vessels were not included. After segmentation, the surface was exported as a stereolithography (STL) file, imported into Meshmixer (v3.5.474; Autodesk, San Francisco, CA, USA), and smoothed [13]. The computational mesh was generated using Ansys Fluent Meshing (2023 R2; Ansys, Canonsburg, PA, USA). We used a hybrid mesh comprising polyhedral elements with seven layers of boundary-fitted prism cells. The first-layer height was 0.05 mm with a growth rate of 1.2, and we confirmed peak y + < 1 in all cases [31]. For each case, we generated low-, medium-, and high-density meshes and performed a mesh-sensitivity analysis on the aortic plane at the distal FET end [32–34]; details are provided in Supplement 1. Across all cases, the absolute differences in WSS between high and medium meshes ranged from 0.000 to 0.0123 Pa, and between medium and low meshes from 0.000 to 0.0131 Pa, indicating mesh convergence. Therefore, the high-density mesh was used for the final simulations to maximize numerical accuracy.

Study 2. A 3D model was constructed using Ansys SpaceClaim (2023 R2; Ansys). Two coaxial tube models were created: (1) an aortic cylinder representing the descending aorta (outer diameter 25 mm, inner diameter 21 mm, length 150 mm), and (2) a stent-graft cylinder (outer diameter 21 mm, inner diameter 19 mm, length 60 mm).

To reduce computational cost, the stent-graft model reproduced the smallest Frozenix configuration (diameter 21 mm, stent length 60 mm). Because the descending aortic diameter is typically reported to range from approximately 18 to 32 mm [35], we selected an aortic model diameter that allowed the stent graft to fit without oversizing. We set the aortic model length to 150 mm. Anatomical measurements report thoracic descending aortic length of approximately 18.4–19.4 cm depending on the definition [35]; thus, 150 mm was considered a physiologically reasonable segment representing part of the thoracic descending aorta.

The stent graft cylinder was placed within the aortic cylinder at four angles—0°, 20°, 40°, and 60°—as illustrated in Fig. S13. Mesh generation and mesh-sensitivity analyses were performed using the same approach as in Study 1 (Supplement 1).

### CFD Simulation

Studies 1 and 2. Blood flow simulations were performed with Ansys Fluent (2023 R2; Ansys) by numerically solving the Navier–Stokes equations under the finite-volume framework. Rigid, no-slip boundary conditions were assumed for the aortic wall. Blood was modeled as a Newtonian fluid with density 1,060 kg/m³ and dynamic viscosity 0.0035 Pa·s [36, 37].

**Fig. 3:**
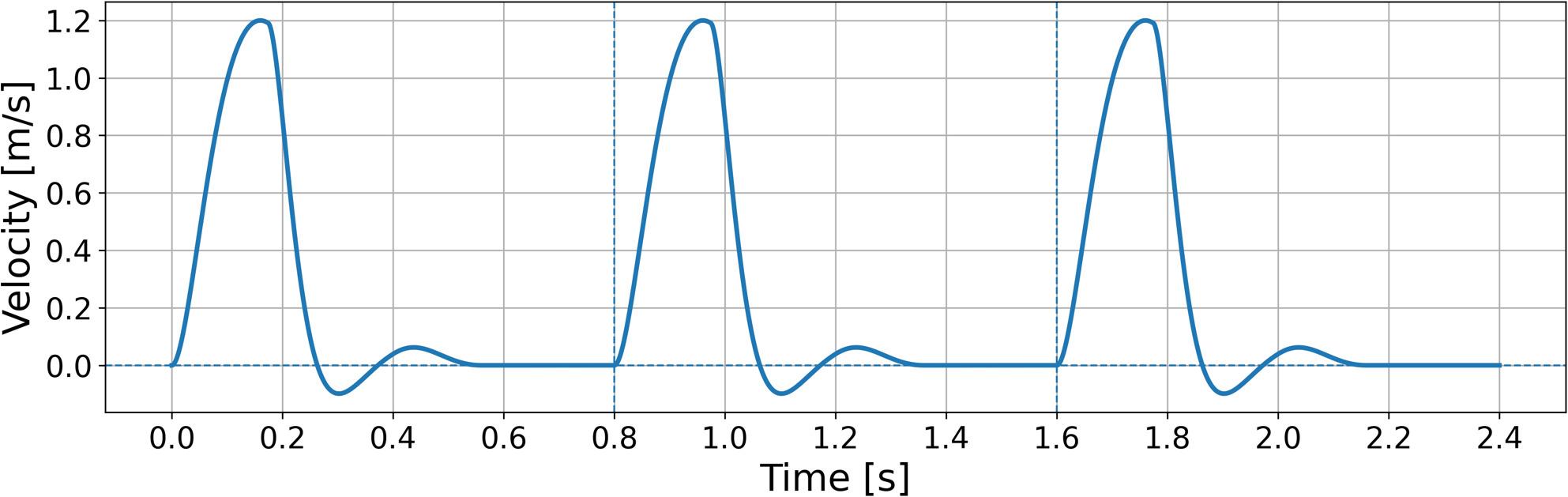
Inlet pulsatile velocity waveform. pulsatile inlet velocity waveform with period T=0.8 s and peak velocity 1.2 m/s was prescribed at the proximal FET inlet

The inlet was defined at the proximal FET, and the outlet at the thoracic descending aorta, positioned approximately two inlet diameters upstream and five outlet diameters downstream from the region of interest around the distal FET [38]. At the inlet, we prescribed a pulsatile velocity waveform with period T = 0.8 s and peak velocity 1.2 m/s (Fig. 3). The waveform was reconstructed as a user-defined function based on 4D-flow MRI–derived velocity profiles reported by Scott et al. [39] and Black et al. [40], representing the distal aortic arch. For mesh-sensitivity analyses, we used a steady inlet condition at the peak velocity of 1.2 m/s. Because our cohort consisted of post-ATAAD surgical patients under strict blood pressure control per guideline recommendations [41], we prescribed a fixed outlet pressure of 100 mmHg (≈ 13,332 Pa) as the representative postoperative target pressure [31]. Given the likelihood of disturbed/transitional flow near the distal FET due to curvature and abrupt geometric change, we used the k–ω SST turbulence model [42, 43]. The time step was 0.01 s. Simulations were run for three cardiac cycles; after confirming convergence by the second cycle, we reported results from the third cycle. We also confirmed mass conservation by comparing inlet and outlet mass flow rates at every time step.

### Hemodynamic parameters

We performed qualitative and quantitative assessments of streamlines, wall shear stress (WSS), time-averaged wall shear stress (TAWSS), and oscillatory shear index (OSI). Because prior work indicates that flow rate/velocity peaks at peak systole and WSS tends to reach its maximum around this phase [44], we evaluated streamlines and instantaneous WSS qualitatively at peak systole. These variables were calculated as follows:

$$\:WSS:\overrightarrow{\tau\:w}=\mu\:\frac{dW}{dl}$$

where $\:l$ is the distance to the vessel wall, $\:W$ is the velocity component parallel to the wall, and $\:\mu\:$ is the viscosity.

WSS represents the instantaneous shear stress acting on the vessel wall. To evaluate shear exposure over the entire cardiac cycle, we computed the time-averaged wall shear stress (TAWSS).

$$\:TAWSS=\frac{1}{T}\int\:\left|\overrightarrow{WSS\left(t\right)}\right|dt$$

OSI characterizes the oscillatory nature of WSS and is computed over one cardiac cycle T: thus, the OSI reflects how much the direction of shear stress changes during a single cardiac cycle [45].

$$\:OSI=\frac{1}{2}\left(1.0-\frac{\left|{\int\:}_{0}^{T}\overrightarrow{\tau\:w}dt\right|}{{\int\:}_{0}^{T}\left|\overrightarrow{\tau\:w}\right|dt}\right)$$

Based on prior studies, we defined low WSS as ≤ 0.4 Pa [46] and high OSI as ≥ 0.2 [47].

### Statistical analysis

All statistical analyses were performed with EZR (Saitama Medical Center, Jichi Medical University, Saitama, Japan), which is a graphical user interface for R (The R Foundation for Statistical Computing, Vienna, Austria) [48].

## Results

### Study 1

#### Statistical analysis

**Fig. 4:**
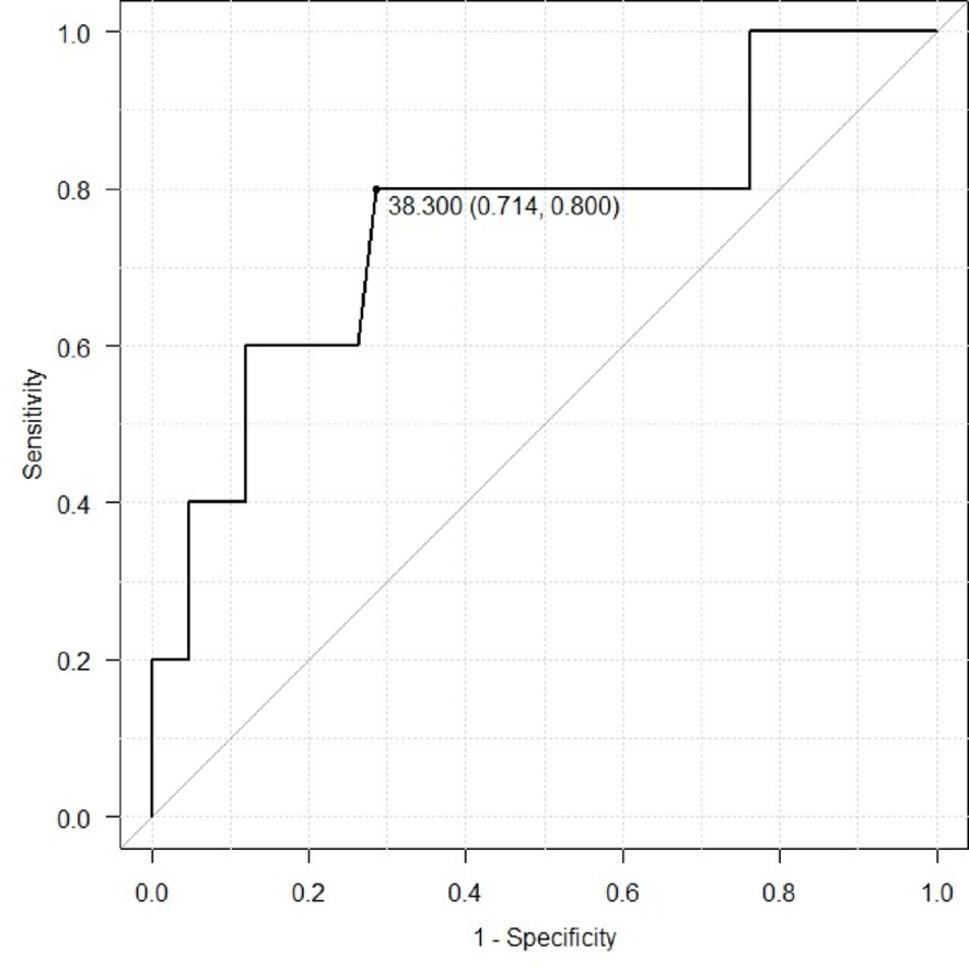
The receiver-operating characteristic curve related to the AS angle and aortic enlargement. AS angle, aorto-stent graft angle

Table 1 summarizes baseline characteristics and compares the enlargement and non-enlargement groups. There were no significant between-group differences in age, sex, or other baseline variables. Operative variables, including FET diameter and length, were also not significantly different; notably, larger diameters (e.g., 31–33 mm) were not preferentially used in the enlargement group. Follow-up duration was not significantly different. The median AS angle was 48.4° in the enlargement group and 28.0° in the non-enlargement group. Mean AS angle was 43.0 ± 15.7° versus 28.8 ± 14.1°, respectively (*p* = 0.041). Table 2 presents logistic regression results for predictors of aortic enlargement, showing a trend toward an association between AS angle and enlargement (*p* = 0.06). Figure 4 shows the ROC curve for AS angle and aortic enlargement with a cutoff value of 38.3° (sensitivity 80%, specificity 71.4%, AUC 0.76), suggesting that AS angle > 38.3° may indicate increased risk of enlargement.

**Table 1:** Comparison of Enlargement and Non-enlargement Groups

Variables	All (*n* = 47)	Enlargement (*n* = 5)	non enlargement (*n* = 42)		*P*-value
Patient backgrounds					
Age (years)	62.1 ± 11.8	67.7 ± 11.4	61.5 ± 11.8	0.274	
Male (sex)	22/47 (46.8%)	3 (60.0%)	19 (45.2%)	0.654	
Hypertension	40/47 (85.1%)	4 (80.0%)	36 (85.7%)	0.571	
Stroke history	1/47 (2.1%)	0 (0.0%)	1 (2.4%)	1.000	
Coronary artery disease	2/47 (4.3%)	1 (20.0%)	1 (2.4%)	0.204	
Renal failure (Cr^a^>1.5 mg/dl)	2/47 (4.3%)	0 (0.0%)	2 (4.8%)	1.000	
Diabetes mellitus	2/47 (4.3%)	0 (0.0%)	2 (4.8%)	1.000	
Hyperlipidemia	16/47 (34.0%)	3 (60.0%)	13 (31.0%)	0.320	
Smoking history	21/47 (44.7%)	3 (60.0%)	18 (42.9%)	0.644	
Emergency surgery	45/47 (95.7%)	4 (80.0%)	41 (97.6%)	0.204	
Operative data					
FET^b^ Diameter					
23 mm	12/47(25.5%)	2 (40.0%)	10 (23.8%)	0.163	
25 mm	18/47(38.3%)	0 (0.0%)	18 (42.9%)		
27 mm	7/47(14.9%)	1 (20.0%)	6 (14.3%)		
29 mm	6/47(12.8%)	2 (40.0%)	4 (9.5%)		
31 mm	2/47(4.3%)	0 (0.0%)	2 (4.8%)		
33 mm	2/47(4.3%)	0 (0.0%)	2 (4.8%)		
FET Length					
90 mm	37/47(78.7%)	5 (100.0%)	32 (76.2%)	0.569	
120 mm	10/47(21.3%)	0 (0.0%)	10 (23.8%)		
Surgical time (min)	377 (IQR:315–451)	438 (IQR:381–493)	369 (IQR:313–449)	0.448	
Circulatory arrest time (min)	46 (IQR:39–55)	54 (IQR:46–56)	46 (IQR:38–54)	0.300	
Cardio-pulmonary bypass time(min)	210 (IQR:178–230)	220 (IQR:212–240)	204 (IQR:178–228)	0.351	
Aortic cross-clamp time(min)	100 (IQR:80–127)	140 (IQR:121–142)	96 (IQR:79–122)	0.129	
Short-term clinical outcome					
Stroke	9/47(19.1%)	1 (20.0%)	8 (19.0%)	1.000	
Paraparesis	1/47(2.1%)	0 (0.0%)	1 (2.4%)	1.000	
Rethoracotomy for bleeding	1/47(2.1%)	0 (0.0%)	2 (4.8%)	1.000	
Permanent dialysis	4/47(8.5%)	1 (20.0%)	3 (7.1%)	0.372	
Deep sternal infection	2/47(4.3%)	0 (0.0%)	2 (4.8%)	1.000	
Long-term clinical outcome					
Follow-up days	1119 (IQR:616–1837)	1569 (IQR:1485–2247)	1109 (IQR:563–1791)	0.147	
Days to remodeling	146 (IQR:70–280)	12 (IQR:8–90)	159 (IQR:96–282)	0.055	
AS Angle^c^ after remodeling(°)	30.3 ± 14.8	43.0 ± 15.7	28.8 ± 14.1	**0.041**	

(a) Creatinine, (b) frozen elephant trunk, (c) Aorto-Stentgraft angle Nominal and categorical variables were compared using the Fisher’s exact test. Continuous variables were compared using a t test or the Mann–Whitney U test. All P-values were 2-sided, with a value < 0.05 considered statistically significant. The enlargement group had a significantly larger AS angle

**Table 2:** Univariate logistic‑regression analysis for predictors of aortic enlargement

Variables	Odds ratio	95% CI	*P*-value
Patient backgrounds			
Age (years)	1.050	0.961–1.15	0.276
Male (sex)	1.820	0.274-12.0	0.536
Hypertension	0.667	0.063–7.03	0.736
Stroke history	-	-	-
Coronary artery disease	10.20	0.533-197.0	0.123
Renal failure (Cr^a^>1.5 mg/dl)	-	-	-
Diabetes mellitus	-	-	-
Hyperlipidemia	3.350	0.498–22.5	0.214
Smoking history	2.000	0.302–13.2	0.472
Emergency surgery	0.098	0.051–1.88	0.123
Operative data			
FET^b^ Diameter	1.050	0.751–1.46	0.786
FET Length	-	-	-
Surgical time (min)	1.000	0.993–1.01	0.499
Circulatory arrest time (min)	1.060	0.955–1.18	0.268
Cardio-pulmonary bypass time(min)	1.010	0.992–1.03	0.297
Aortic cross-clamp time(min)	1.020	0.992–1.04	0.195
Short-term clinical outcome			
Stroke	1.060	0.104–10.8	0.959
Paraparesis	-	-	-
Rethoracotomy for bleeding	-	-	-
Permanent dialysis	3.250	0.270-39.0	0.353
Deep sternal infection	-	-	-
Long-term clinical outcome			
Follow-up days	1.000	1.00–1.00	0.163
Days to remodeling	0.994	0.985-1.00	0.208
AS Angle^c^ after remodeling(°)	1.080	0.997–1.17	0.060

(a) Creatinine, (b) frozen elephant trunk, (c) Aorto-Stentgraft angle

A hyphen indicates that the variable could not be analysed because no events occurred in one group. Although no variable reached conventional statistical significance, the AS angle showed a trend toward association (*P* = 0.06)

#### CFD analysis of patient-specific models

**Fig. 5:**
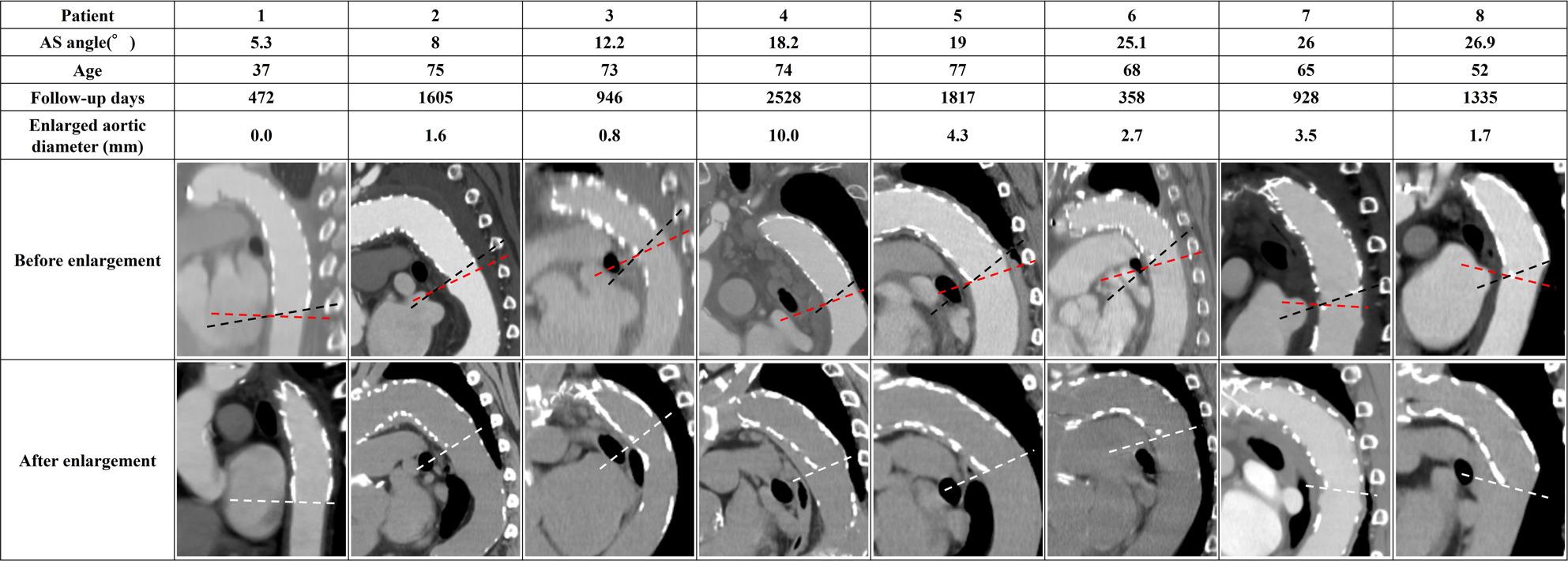
Patient-specific AS angles and CT views (Patients 1–8). AS angle is formed by the red and black dashed lines. Enlarged aortic diameter was defined as the difference between the post-enlargement plane (white dashed line) and the pre-enlargement plane (red dashed line) at the distal FET level. AS angle, aorto-stent graft angle

**Fig. 6:**
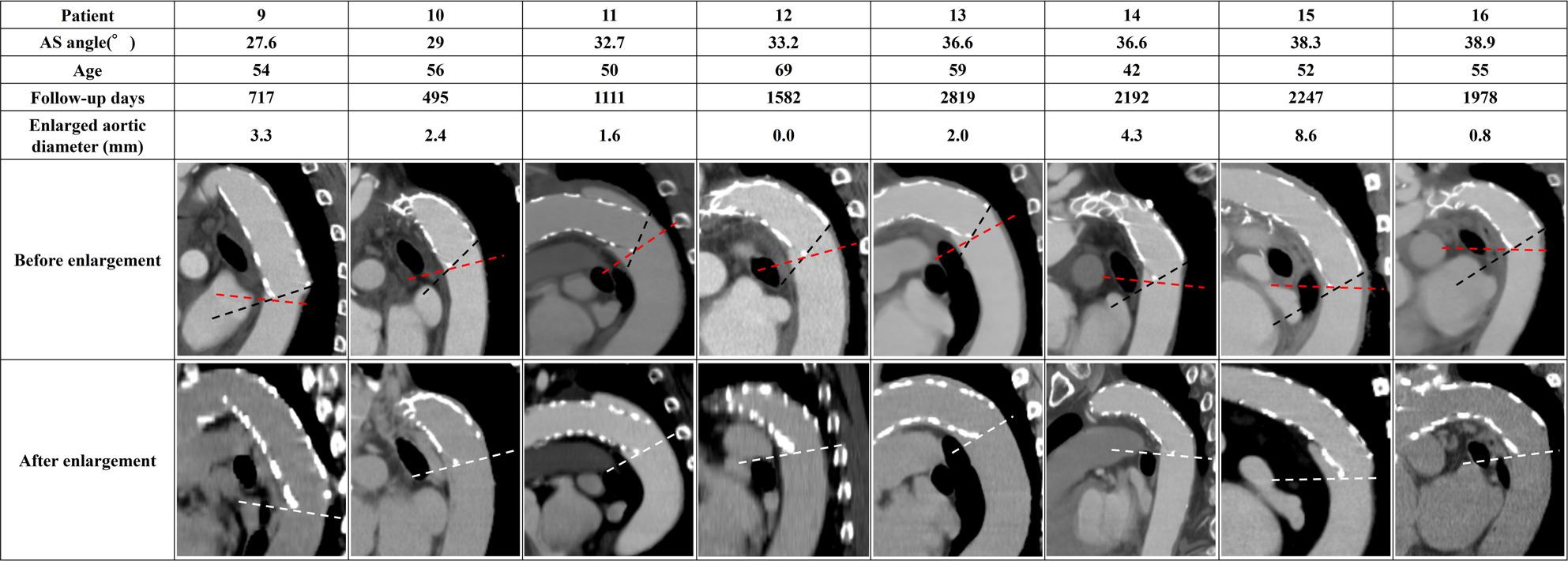
Patient-specific AS angles and CT views (Patients 9–16)

**Fig. 7:**
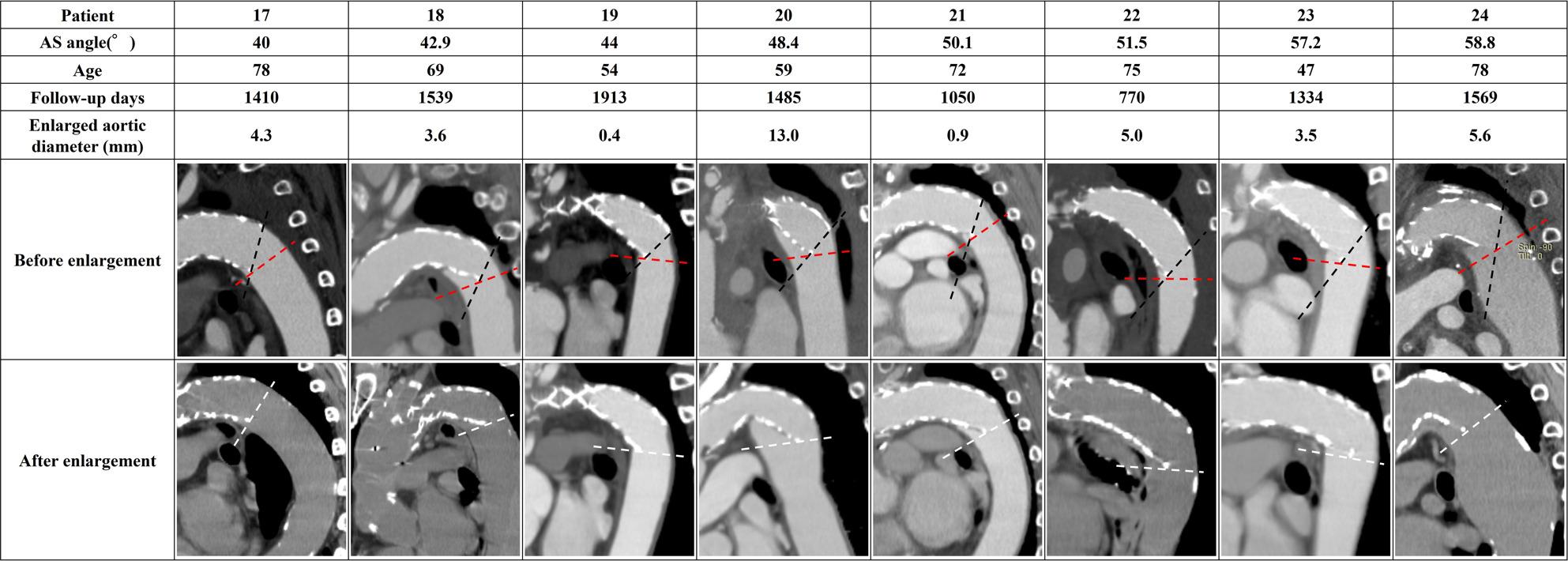
Patient-specific AS angles and CT views (Patients 17–24)

**Fig. 8:**
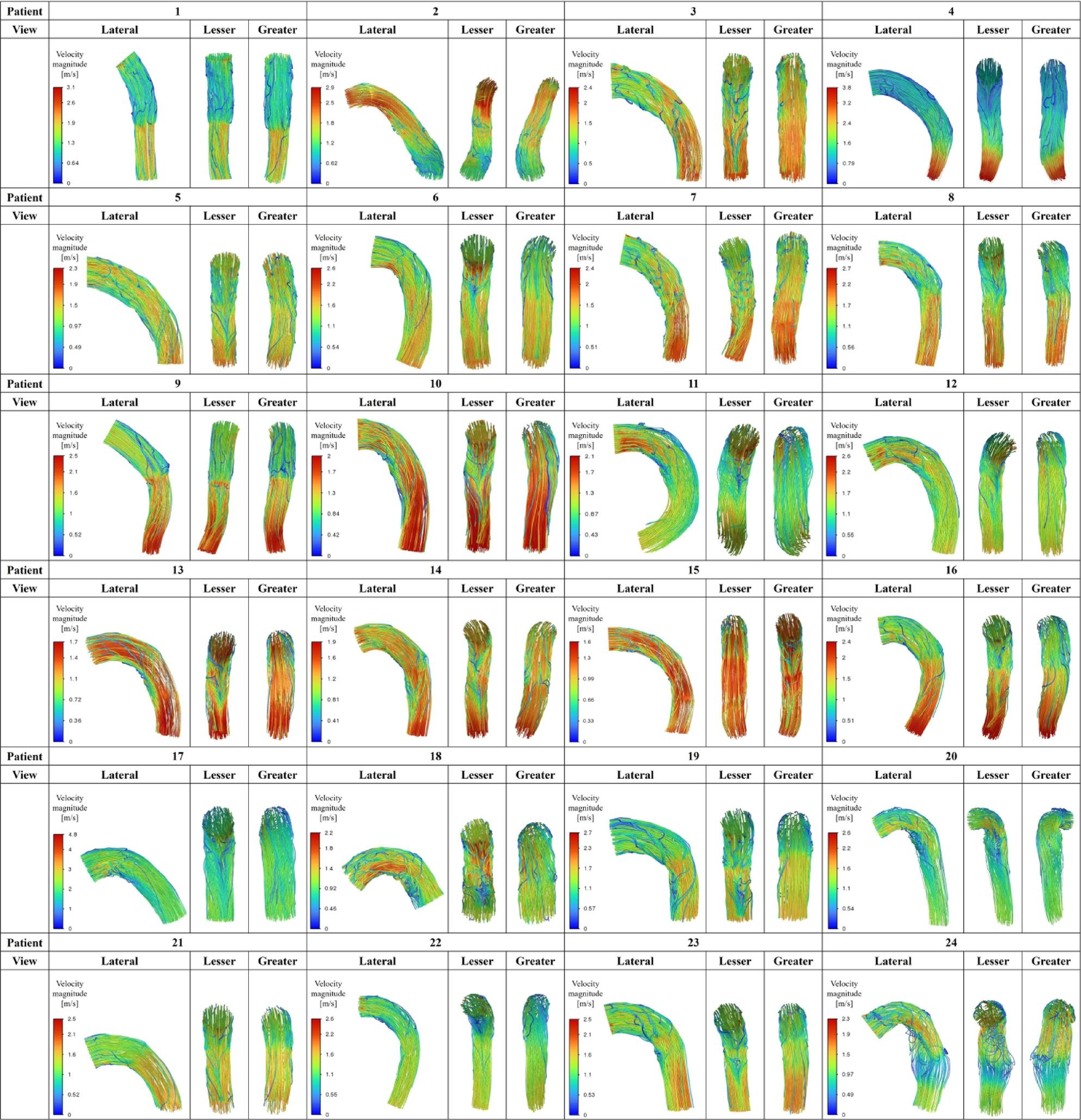
Streamlines at peak systole (Study 1) treamlines at peak systole for all 24 patient-specific cases. Disturbed/recirculating flow along the lesser curvature was observed in some cases (Patients 11, 18, 19, 20, 22, 23, and 24)

**Fig. 9:**
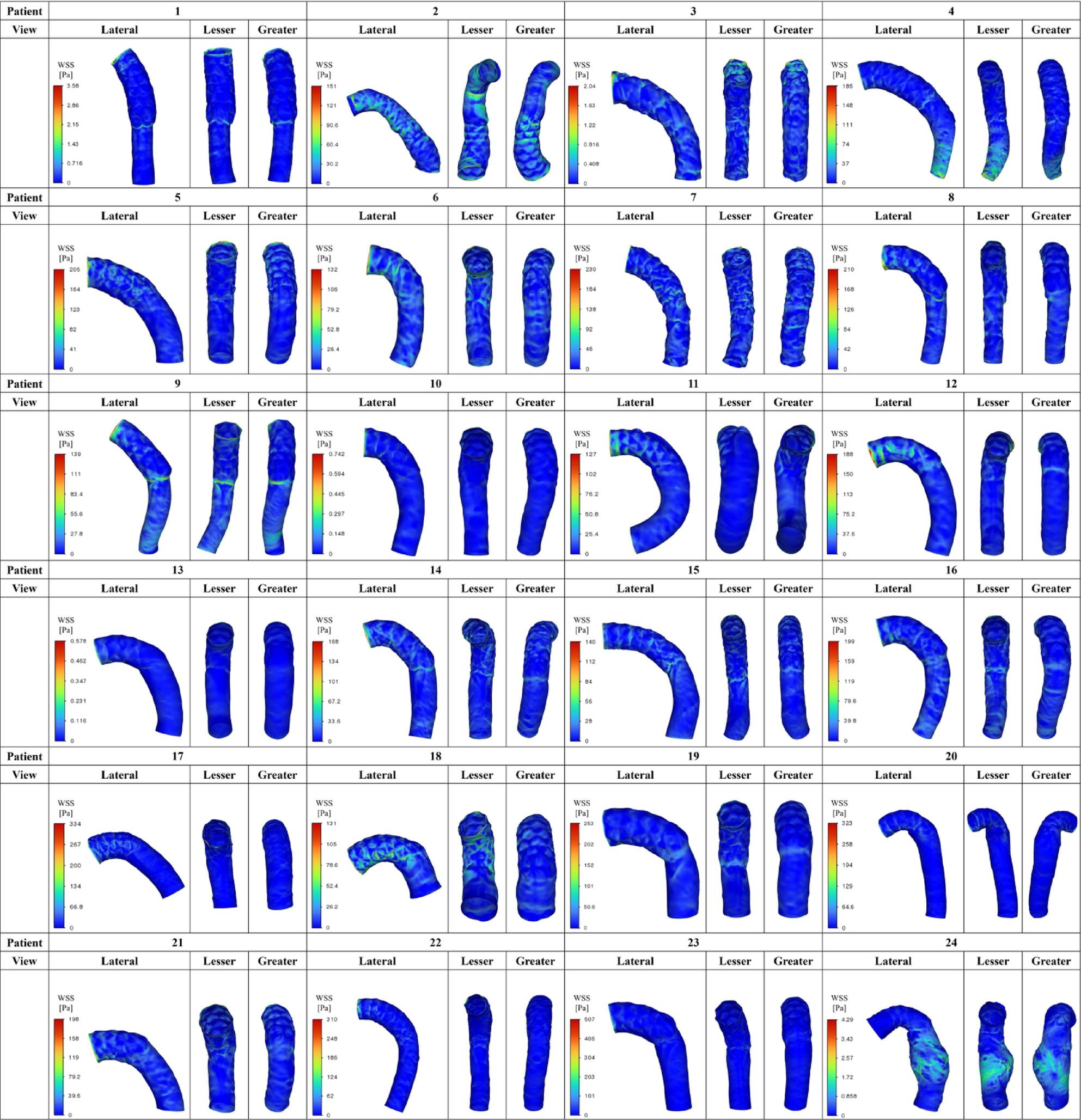
Peak-systolic WSS (Study 1) Peak-systolic wall shear stress (WSS) in all 24 cases. High-WSS regions on the aortic wall distal to the stent graft were observed in Patients 9, 12, 14, 15, 16, and 19

Figures 5, 6 and 7 summarize all 24 CFD cases ordered by AS angle. Based on the ROC-derived cutoff, high-risk cases with AS angle ≥ 38.3° corresponded to Patients 15–24. AS angle was defined by the angle formed by the black and red dashed lines, and quantitative hemodynamic measurements were obtained at the red dashed line plane. Qualitative results using streamlines and contour maps are shown first. Figure 8 shows streamlines at peak systole: Patients 11, 18, 19, 20, 22, 23, and 24 demonstrated disturbed/recirculating flow along the lesser curvature (agreement with the high-risk classification: 85.7% [6/7]). Figure 9 shows WSS at peak systole: Patients 9, 12, 14, 15, 16, and 19 exhibited high-WSS regions on the aortic wall distal to the stent graft (high-risk agreement: 50.0% [3/6]). Figure 11 shows TAWSS: Patients 10 and 13 had diffusely low WSS, whereas Patients 1, 8, 9, 12, 14, 15, 16, 18, 19, 21, and 23 showed high-TAWSS regions on the aortic wall distal to the stent graft (high-risk agreement: 54.5% [6/11]). Figure 11 shows OSI: Patients 3, 5, 6, 10, 13, 14, 15, 17, 18, 19, 20, 21, 22, and 24 had continuous high-OSI regions along the lesser curvature (high-risk agreement: 57.1% [8/14]). Conversely, some cases (e.g., Patients 3, 14, 19, 21, 23) showed continuous low-OSI regions along the greater curvature (high-risk agreement: 60.0% [3/5]).

Supplement 2 provides quantitative measurements at the evaluation plane. Across all 24 patients, median (IQR) mean WSS was 0.02712 (0.02201–0.03706) Pa and peak WSS was 0.12262 (0.08989–0.18105) Pa, indicating low WSS even at peak systole. Mean TAWSS was 0.02415 (0.01769–0.03542) Pa and peak TAWSS was 0.02730 (0.02004–0.04069) Pa, also low. In contrast, mean OSI was 0.49713 (0.49681–0.49753) and peak OSI was 0.49717 (0.49685–0.49758), indicating high OSI. Figure 12 shows Pearson correlations between AS angle and each index. Although not statistically significant, peak WSS and peak TAWSS tended to show negative correlations (*p* = 0.1020 and *p* = 0.1197, respectively).

### Study 2: CFD analysis of idealized models

Figure 13 shows the 3D aorta–stent graft models at AS angles of 0°, 20°, 40°, and 60°. Based on the ROC-derived cutoff (38.3°), the 40° and 60° models correspond to the high-risk range. Figure 14 shows streamlines at peak systole. With increasing angle, velocity increased along the greater curvature immediately distal to the stent graft, while disturbed/recirculating flow emerged along the lesser curvature. At 20°, peak velocity was approximately 1.3 m/s; at ≥ 40°, peak velocity increased to ~ 2.1 m/s, exceeding the prescribed inlet peak of 1.2 m/s. At 60°, peak velocity increased to ~ 4.0 m/s. Figure 15 shows peak-systolic WSS: greater-curvature WSS increased with angle, reaching ~ 100 Pa, whereas lesser-curvature WSS remained low. Figure 16 shows TAWSS: greater-curvature TAWSS increased with angle and was particularly high at the lateral wall distal to the stent graft; lesser-curvature TAWSS remained low except for a small band-like increase in the 60° model. Figure 17 shows OSI: with increasing angle, contrast intensified between high OSI on the lesser curvature and low OSI on the greater curvature; this contrast became prominent at angles > 40°. With larger angles, the band-like high-OSI region on the lesser curvature started more proximally.

Supplement 2 provides quantitative measurements at the evaluation plane for the four models. Median (IQR) mean WSS was 0.03335 (0.01923–0.05481) Pa and peak WSS was 0.17759 (0.08091–0.32845) Pa, indicating low WSS even at peak systole. Mean TAWSS was 0.03471 (0.01864–0.06003) Pa and peak TAWSS was 0.03932 (0.02052–0.06908) Pa, also low. Mean OSI was 0.49581 (0.49564–0.49596) and peak OSI was 0.49588 (0.49570–0.49603), indicating high OSI. Figure 2 shows Pearson correlations between AS angle and hemodynamic indices. Mean and peak WSS were strongly correlated with AS angle (*r* = 0.965, *p* = 0.0349; *r* = 0.964, *p* = 0.0355). Mean and peak TAWSS were also strongly correlated with AS angle (*r* = 0.963, *p* = 0.0373; *r* = 0.962, *p* = 0.0375).

## Discussion

In this study, we hypothesized that a steep angle between the distal FET and the native aorta (AS angle) may be associated with subsequent aortic enlargement. First, in Study 1 we performed statistical analyses and CFD (patient-specific models) in patients who achieved favorable early remodeling after TAR with FET. AS angle was significantly larger in the enlargement group (43.0 ± 15.7°) than in the non-enlargement group (28.8 ± 14.1°, *p* = 0.041; Table 1). Logistic regression suggested a trend toward an association between AS angle and enlargement (*p* = 0.06; Table 2). ROC analysis yielded a cutoff of 38.3° with sensitivity 80% and specificity 71.4% (AUC = 0.76). Defining AS angle ≥ 38.3° as “high risk” corresponded to Patients 15–24 in Study 1 and the 40°/60° models in Study 2. Because subtle findings may not appear at small angles, we also assessed how often qualitative CFD findings were observed in high-risk cases (i.e., agreement with the risk classification).

In Study 1 (patient-specific models), qualitative streamline assessment showed lesser-curvature flow disturbance in 85.7% of high-risk cases. High-WSS regions on peak-systolic contours were observed in 50.0%, high-TAWSS regions in 54.5%, continuous high-OSI regions along the lesser curvature in 57.1%, and continuous low-OSI regions along the greater curvature in 60.0%. Overall, the expected qualitative findings tended to appear in high-risk cases. In quantitative assessments at the evaluation plane (distal to the stent graft), both mean and peak WSS/TAWSS were low, whereas OSI was high.

Study 2 (idealized models) showed that as AS angle increased, velocity immediately distal to the stent graft increased along the greater curvature and disturbed flow emerged along the lesser curvature. Peak velocity was ~ 1.3 m/s at 20° and increased to ~ 2.1 m/s at ≥ 40°. With increasing angle, greater-curvature WSS and TAWSS increased and the low-OSI contrast on the greater curvature became more pronounced. In contrast, lesser-curvature WSS and TAWSS remained low and high-OSI contrast increased; at > 40°, the contrast between low OSI on the greater curvature and high OSI on the lesser curvature became striking. These findings suggest that high-angle configurations produce more prominent hemodynamic abnormalities, consistent with Study 1. Pearson correlation analyses showed that WSS and TAWSS increased significantly with increasing AS angle, consistent with the qualitative CFD findings. Taken together, the statistical and CFD results from Study 1 and the idealized-model results from Study 2 suggest that increasing AS angle may promote lesser-curvature flow disturbance, low WSS/low TAWSS, and high OSI, while increasing greater-curvature WSS/TAWSS—potentially contributing to subsequent aortic enlargement.

**Fig. 10:**
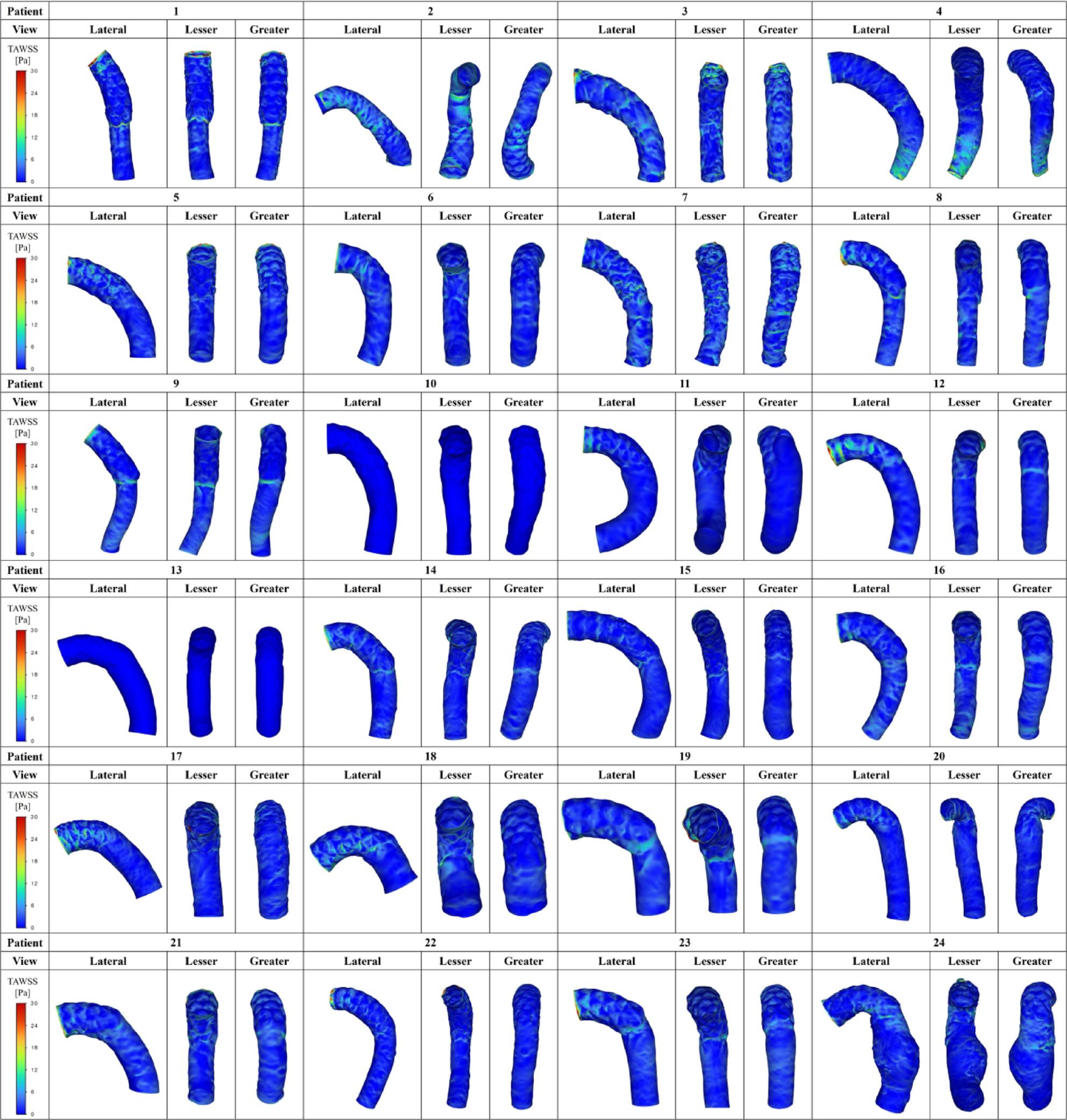
TAWSS distribution (Study 1). Time-averaged wall shear stress (TAWSS) in all 24 cases. Patients 10 and 13 showed diffusely low WSS. High-TAWSS regions distal to the stent graft were observed in Patients 1, 8, 9, 12, 14, 15, 16, 18, 19, 21, and 23

However, some patients did not develop enlargement despite unfavorable hemodynamic indices. Although not statistically significant, the median follow-up was longer in the enlargement group (1569 days) than in the non-enlargement group (1109 days), raising the possibility that some non-enlargement cases may enlarge with longer follow-up. Other protective factors (e.g., aggressive blood-pressure control, smoking cessation) may also have contributed, but the mechanisms remain unclear and warrant further investigation.

**Fig. 11:**
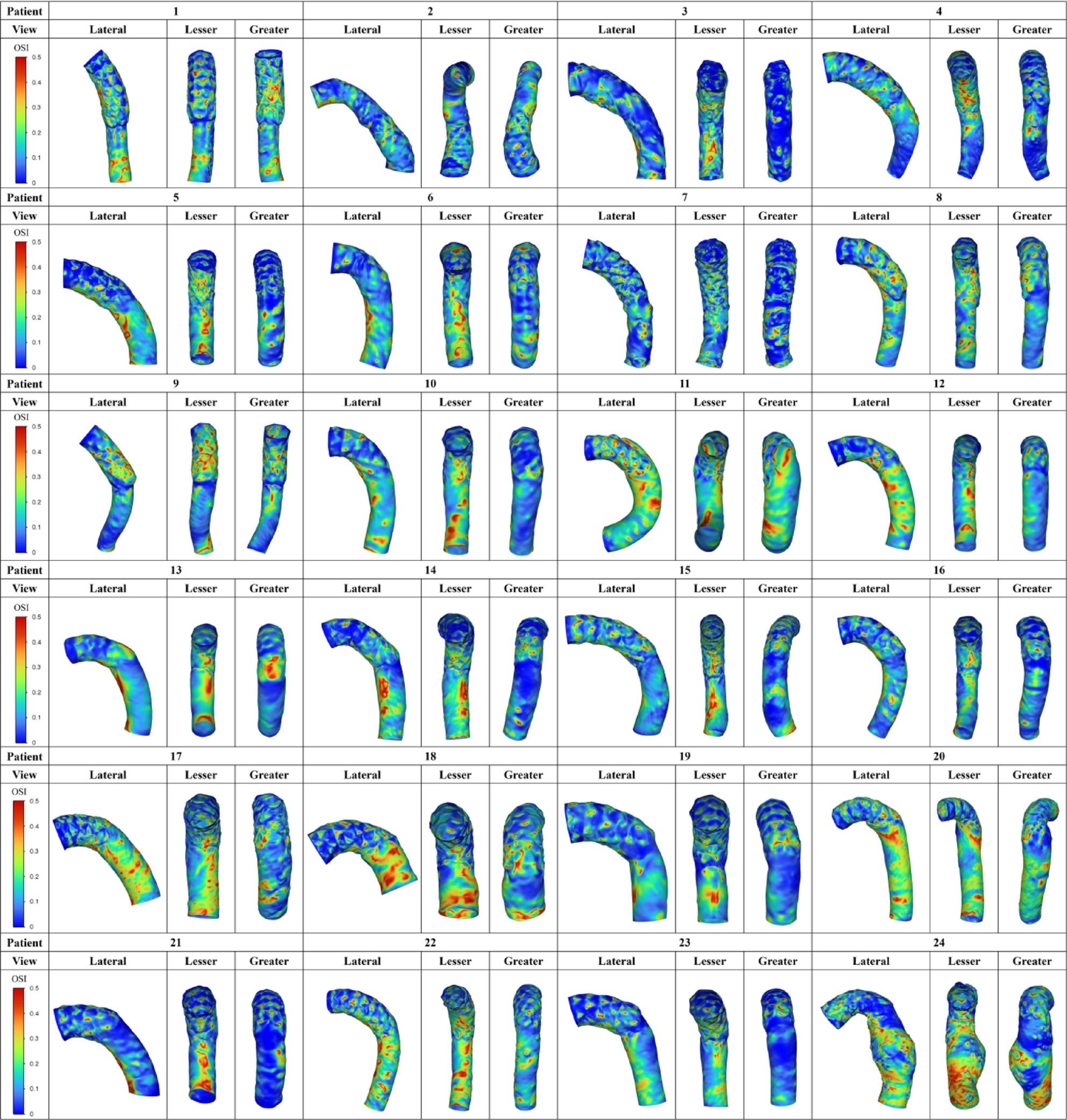
OSI distribution (Study 1). Oscillatory shear index (OSI) in all 24 cases. Continuous high-OSI regions along the lesser curvature were observed in Patients 3, 5, 6, 10, 13, 14, 15, 17, 18, 19, 20, 21, 22, and 24

In this study, the aortic wall was treated as rigid and fluid–structure interaction (FSI) was not considered. Zhu et al. compared FSI and rigid-wall models after type A dissection surgery and reported that, compared with rigid walls, FSI produced lower velocities and WSS and increased low-TAWSS regions—suggesting that rigid-wall assumptions may underestimate low-WSS/low-TAWSS–related risk [49]. Conversely, Mendez et al. evaluated rigid versus FSI models in ascending aortic aneurysm and suggested that if the aneurysm wall is stiff, differences in WSS between rigid and FSI simulations may be smaller [50]. Because our quantitative evaluation region was near the stent graft, the wall may be relatively stiff, making the rigid-wall approximation more plausible; nevertheless, underestimation of low-WSS–related risk remains possible. Accordingly, while we observed an association between high AS angle and low WSS, absolute WSS values may be underestimated.

**Fig. 12:**
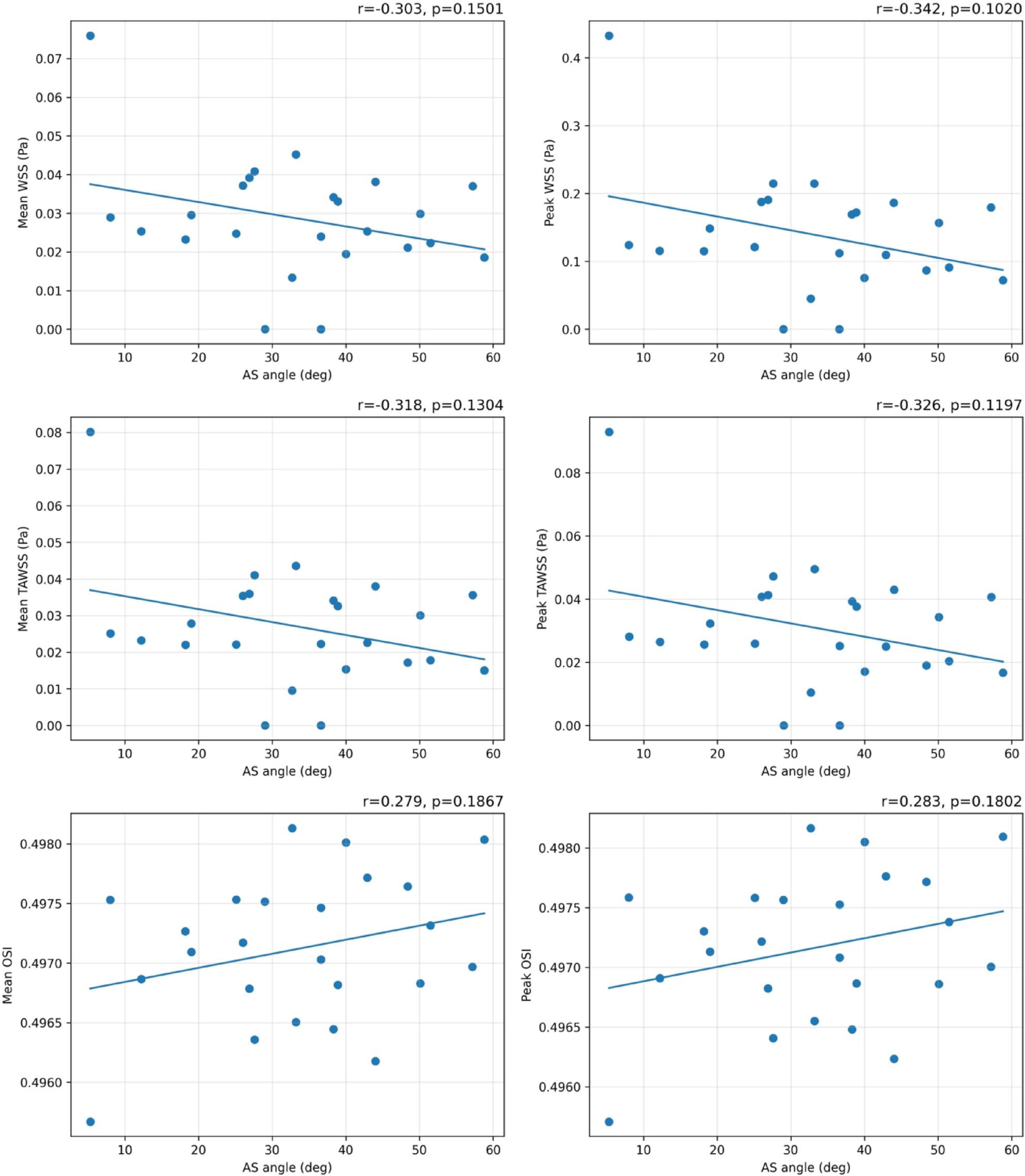
Pearson correlations between AS angle and indices. Pearson correlation analysis between AS angle and mean/peak WSS, TAWSS, and OSI in 24 patient-specific cases. Each panel shows correlation coefficient r and p value

When selecting the Frozenix stent graft, most studies have advised against oversizing its diameter to reduce the risk of SINE [7, 51], whereas others note that its distal end should be located above the aortic valve level to avoid spinal cord injury [52]. However, few studies provide concrete evidence on the ideal graft length to minimize AS angle. Based on our findings, we recommend measuring AS angle during preoperative CT planning and selecting an FET length that may minimize this angle. In our institution, we deploy the FET from zone 2. Preoperatively, we generate a sagittal CT view from the arch to the descending aorta, measure the distance from just proximal to the left subclavian artery to the descending aorta, and choose an FET length that becomes as parallel as possible to the descending aorta while remaining above the aortic valve level. In our cohort of 47 successfully remodeled patients, incomplete paraplegia occurred in 1 patient (2.1%; Table 1), which we consider acceptable compared with a meta-analysis of > 3,000 patients reporting a spinal cord ischemia rate of 4.7% [53].

**Fig. 13:**
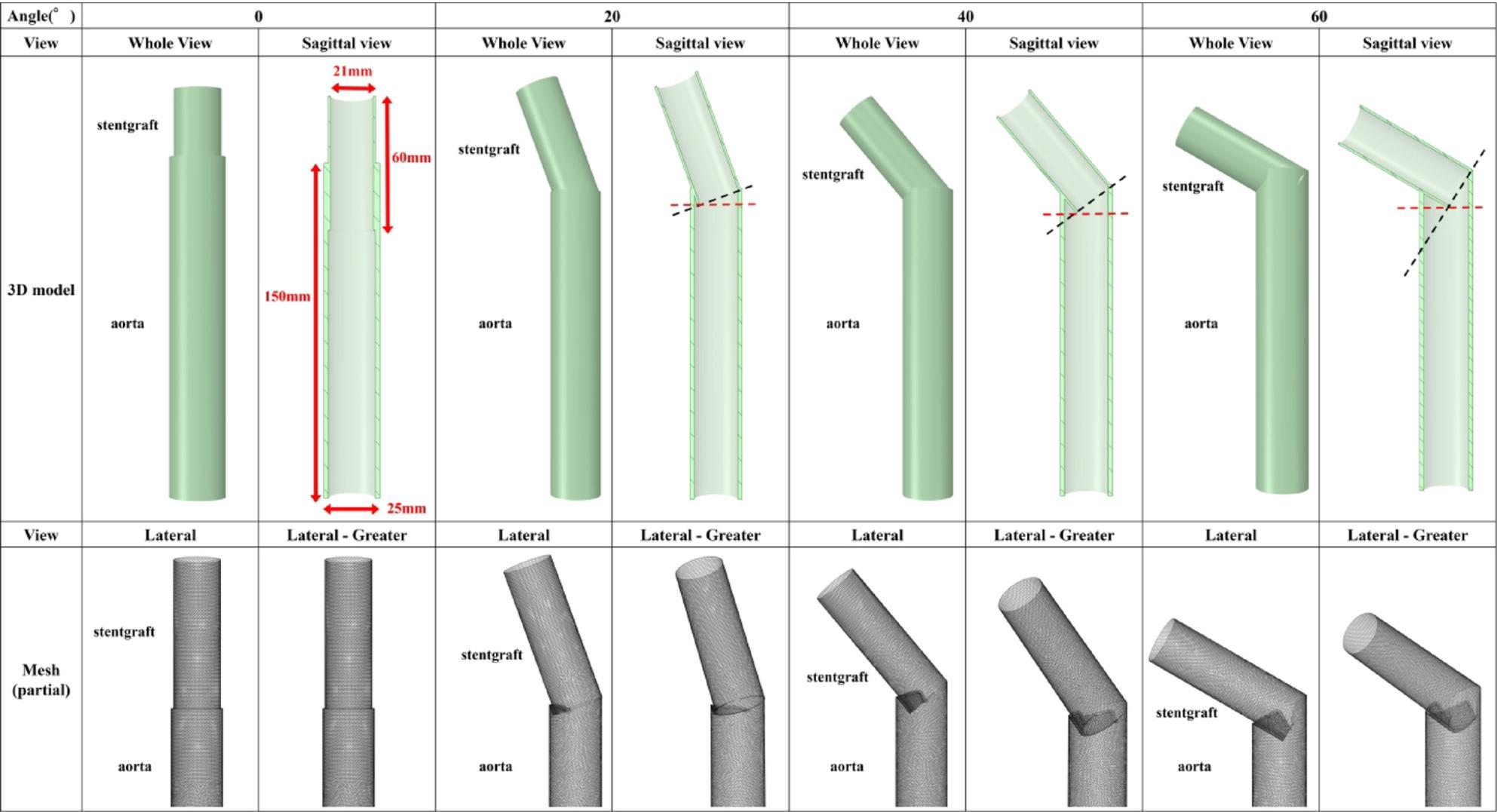
Idealized models and mesh (Study 2). Three-dimensional models were constructed: one representing the aorta and the other representing the stent graft. A CFD analysis was then conducted. The stent graft cylinder was positioned within the aortic cylinder at 0°, 20°, 40°, and 60°

If an unfavorable (i.e., large) AS angle is unavoidable, even if initial remodeling is good, clinicians should remain vigilant for possible later reexpansion. If such reexpansion is detected early, preemptive thoracic endovascular aortic repair could be a feasible option because further enlargement would require more extensive and invasive procedures, such as thoracoabdominal aortic repair.

**Fig. 14:**
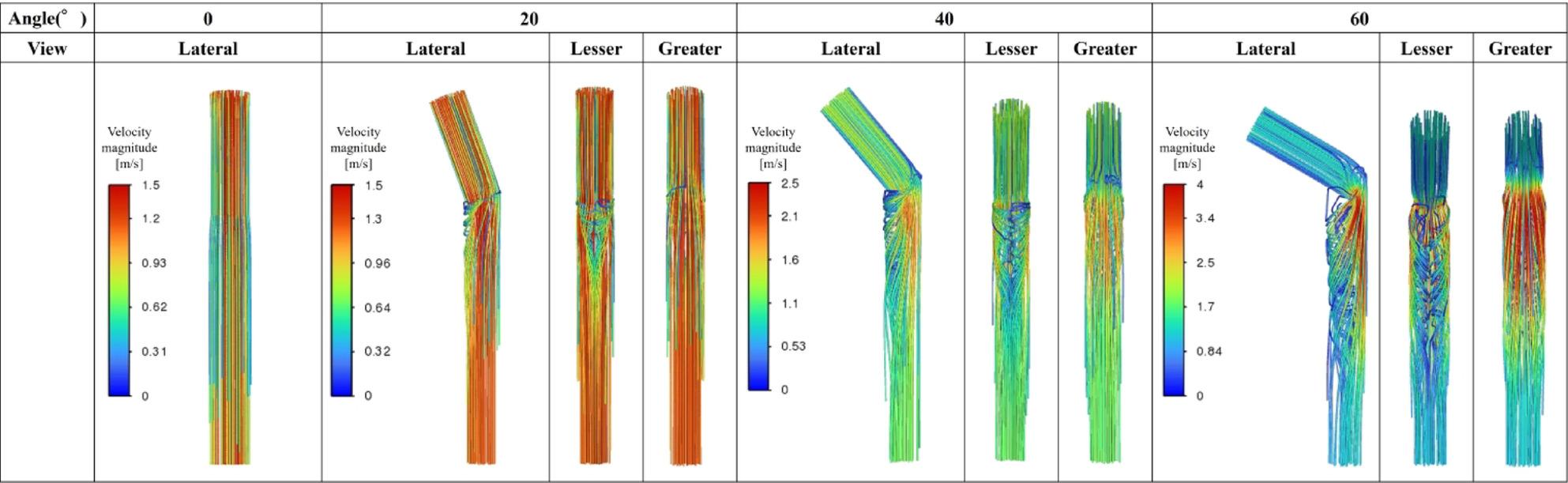
Streamlines at peak systole (Study 2) . With increasing AS angle, velocity increased along the greater curvature immediately distal to the stent graft, while disturbed flow appeared along the lesser curvature. Peak velocity was ~1.3 m/s at 20°, ~2.1 m/s at ≥40°, and ~4.0 m/s at 60°

Regarding device modifications to reduce AS angle, a product called J Graft Frozenix Partial ET (Japan Lifeline, Tokyo, Japan) has been available in Japan since 2023. This device adds a 20-mm fabric-only skirt at the distal end of the Frozenix stent, which may help the graft conform to natural curvature [54]. However, because the device was approved recently and available lengths are limited (stent portion 60 mm only), its indications remain limited and whether it truly improves conformability requires further study.

**Fig. 15:**
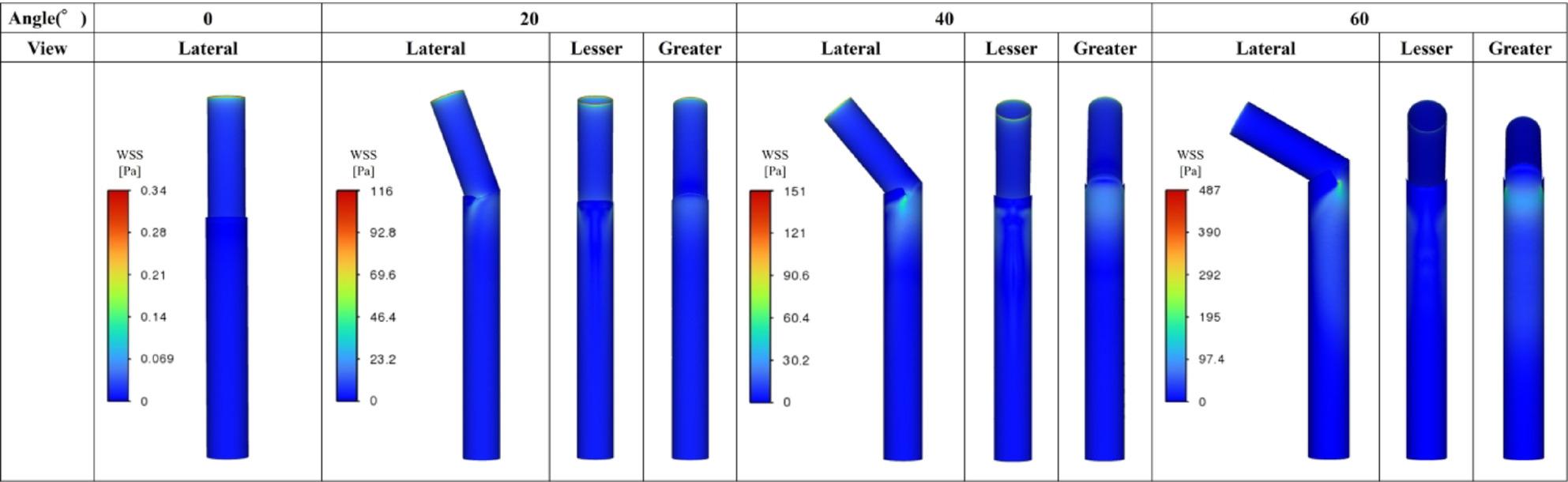
Peak-systolic WSS (Study 2). Greater-curvature WSS increased with angle, reaching ~100 Pa, whereas lesser-curvature WSS remained low

## Conclusion

A larger AS angle may be associated with enlargement of the distal aorta after FET. Larger and more precise studies are needed to validate these findings.

**Fig. 16:**
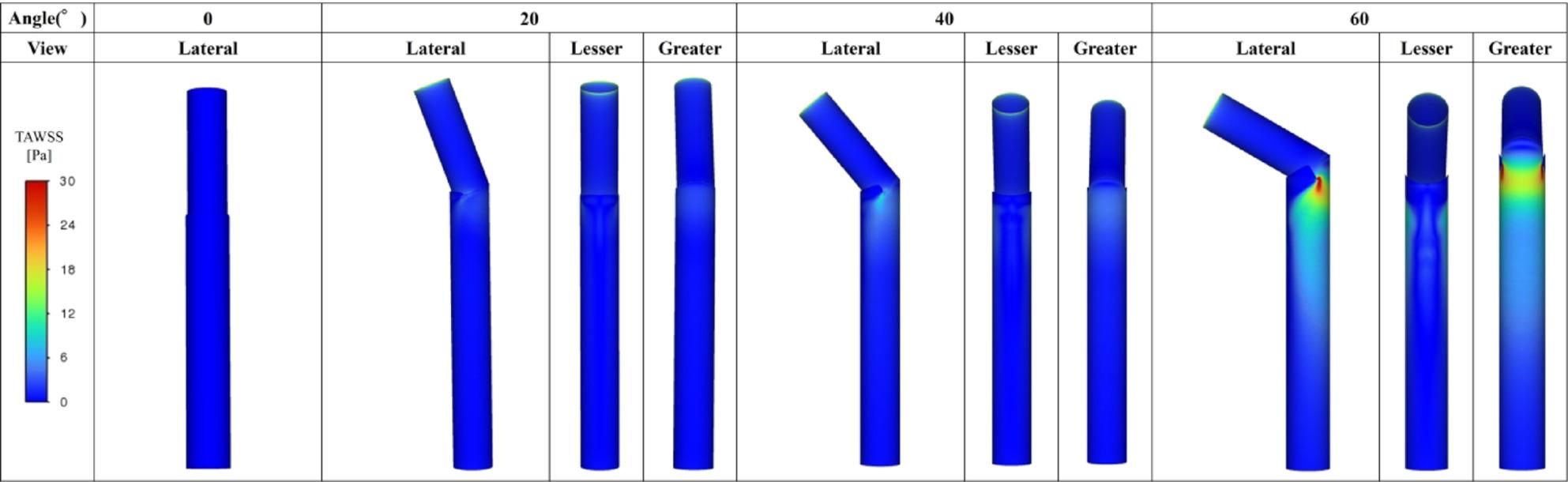
TAWSS distribution (Study 2). Greater-curvature TAWSS increased with angle and was highest distal to the stent graft; lesser-curvature TAWSS remained low except for a small band-like increase at 60°

## Limitations

This retrospective study included a small cohort because the complication of interest is relatively rare, resulting in statistical limitations. Because we used idealized CFD assumptions (rigid wall and simplified boundary conditions), clinical generalizability may be limited. In Study 1, patients without contrast-enhanced CT at the time successful remodeling was confirmed were excluded for CFD, which may have introduced selection bias (e.g., exclusion of patients with impaired renal function). Because low WSS and high OSI did not always lead to enlargement, additional factors and longer follow-up are needed. In Study 2, the aorta and graft were modeled as concentric cylinders, ignoring patient-specific curvature, and the stent structure was not explicitly modeled (smooth cylinder approximation). Future work should incorporate more realistic anatomy and detailed device structure.

**Fig. 17:**
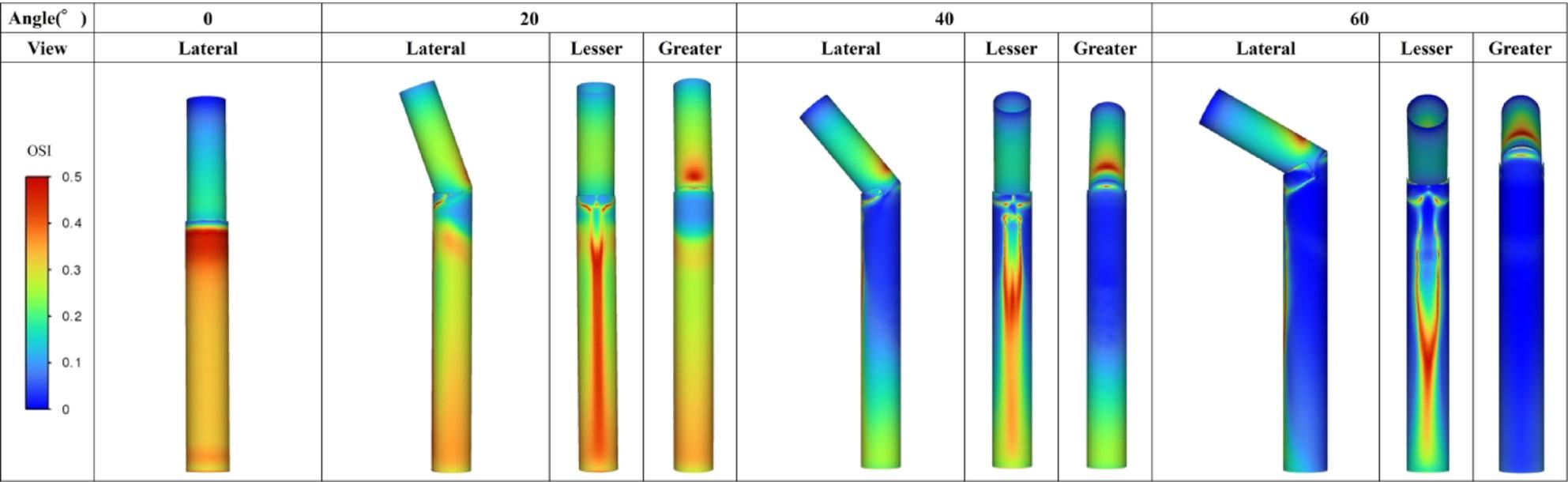
OSI distribution (Study 2). With increasing angle, contrast intensified between high OSI on the lesser curvature and low OSI on the greater curvature; this became prominent at angles >40°

**Fig. 18:**
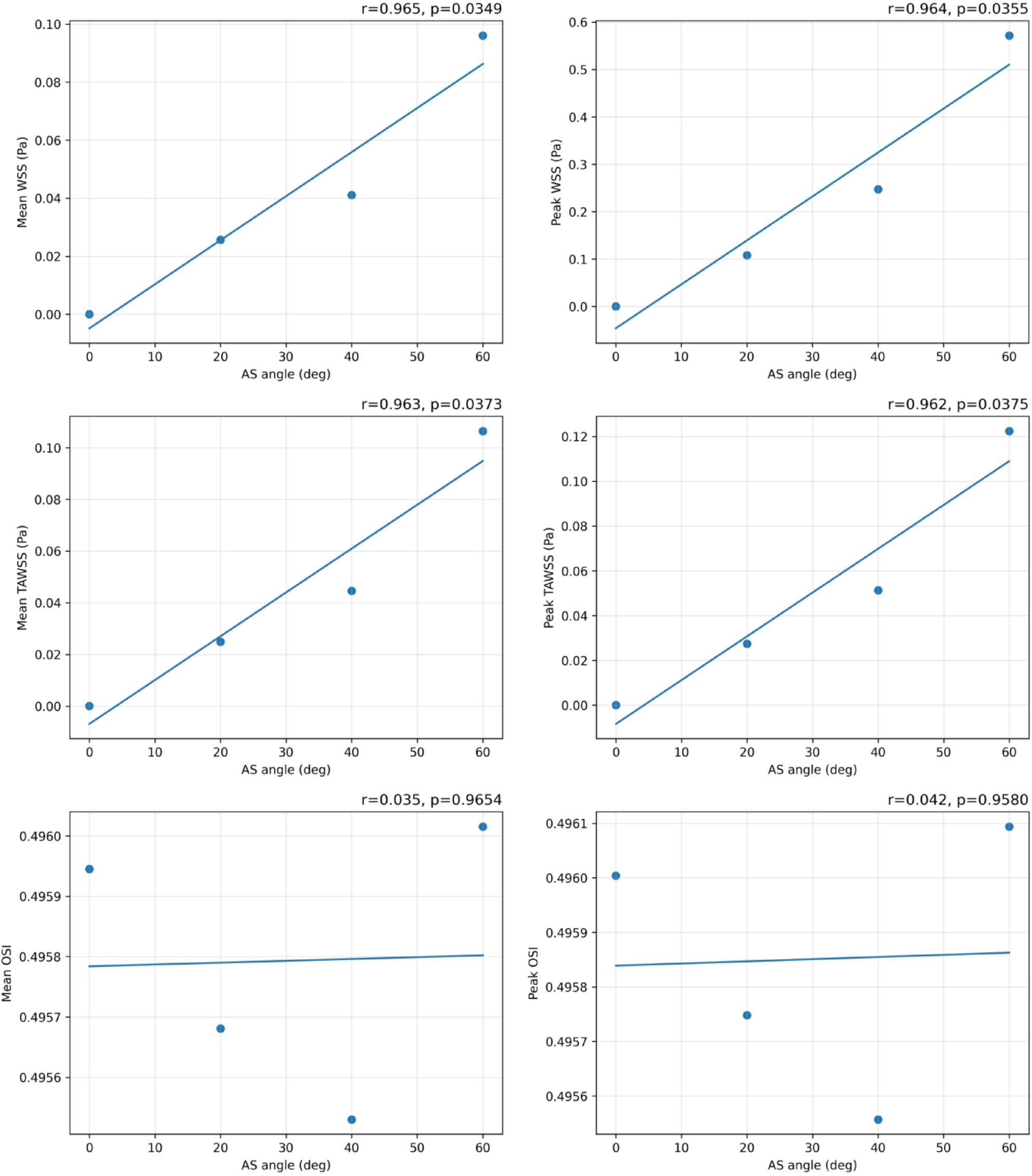
Pearson correlations between AS angle and indices. Pearson correlation analysis between AS angle and mean/peak WSS, TAWSS, and OSI across the four idealized models. Each panel shows r and p value

## Supplementary Information

Below is the link to the electronic supplementary material.

Supplementary Material 1: Supplementary Table 1. Mesh sensitivity analysis and mesh quality metrics

Supplementary Material 2: Supplementary Table 2. Hemodynamic measurements for all cases

## Data Availability

No datasets were generated or analysed during the current study.
